# The effects of a set amount of regular maternal exercise during pregnancy on gut microbiota are diet-dependent in mice and do not cause significant diversity changes

**DOI:** 10.7717/peerj.14459

**Published:** 2022-12-02

**Authors:** Xia Duan, Jingjing Xu, Ping Yang, Xinyuan Liang, Zichun Zeng, Huijuan Luo, Xiaomei Tang, Xin Wu, Xiaomin Xiao

**Affiliations:** 1 Department of Obstetrics and Gynecology, The First Affiliated Hospital of Jinan University, Guangzhou, China; 2 Guangdong Medical Laboratory Animal Center, Foshan, China

**Keywords:** Gut microbiota, Microbiome, Exercise, High-fat diet, Pregnant mice

## Abstract

**Background:**

Diet and exercise can affect the gut microbiota (GM); however, the effects of the same amount of exercise on gut microbiota changes in people on a low-fat diet (LFD) and high-fat diet (HFD) during pregnancy are unknown. Do different nutritional conditions respond equally to exercise intervention? This study aimed to investigate the effects of regular maternal exercise during pregnancy on the GM in mice fed different diets during pregnancy.

**Methods:**

Six-week-old nulliparous female KunMing mice were fed either a HFD or LFD before and during pregnancy. Each group of mice were then randomly divided into two groups upon confirmation of pregnancy: sedentary (HFD or LFD; *n* = 4 and 5, respectively) and exercised (HFDex or LFDex, *n* = 5 and 6, respectively). Mice were sacrificed on day 19 of gestation and their colon contents were collected. We then performed 16S rDNA gene sequencing of the V3 and V4 regions of the GM.

**Results:**

The pregnancy success rate was 60% for LFDex and 100% for HFDex. Both Chao1 and Simpson indices were not significantly different for either LFD vs. LFDex or HFD vs. HFDex. *Desulfobacterota*, *Desulfovibrionia Desulfovibrionales*, *Desulfovibrionaceae*, *Desulfovibrio*, *Coriobacteriia*, *Coriobacteriales,* and *Eggerthellaceae* were markedly decreased after exercise intervention in LFDex vs. LFD, whereas *Actinobacteria*, *Bifidobacteriales*, *Bifidobacteriaceae*, *Bifidobacterium,* and *Bifidobacterium pseudolongum* were significantly increased in LFDex vs. LFD. Furthermore, decreased *Peptostreptococcales-Tissierellales* and *Peptostreptococcaceae* and increased *Bacteroides dorei* were identified in the HFDex vs. HFD group. *p_Desulfobacterota, c_Desulfovibrionia, o_Desulfovibrionales, f_Desulfovibrionaceae* and *g_Desulfovibrio* were markedly decreased in the LFDex group vs. HFDex group.

**Conclusions:**

Our data suggested that quantitative maternal exercise during pregnancy resulted in alterations in GM composition, but did not significantly change the diversity of the GM. These findings may have important implications when considering an individual’s overall diet when recommending exercise during pregnancy.

## Introduction

The human gut microbiota (GM) plays a crucial role in host health (Monda et al., 2017). Through the production of various metabolites and active substances, the GM participates in the development of the digestive, immune, neurological, and other systems of the host and maintains homeostasis of the internal and external environments (Charbonneau et al., 2016). Substantial studies have shown that the composition of the GM can be influenced by various factors, such as antibiotic use, lifestyle, genetics, and aging, and that it varies in different physiological states (Cresci & Bawden, 2015; Monda et al., 2017; Motiani et al., 2020). During pregnancy, hormonal, immune, and metabolic changes occur in the maternal body. These physiological changes lead to changes in the environment in which human microbial parasites reside, which in turn affects the colonization of the GM in pregnant women (Edwards et al., 2017). The GM profile changes as pregnancy progresses (Koren et al., 2012). The GM changes in women in their third pregnancy trimester, as compared to non-pregnant women. An increase in β-diversity among individuals, and reduced relative microbial abundance and α-diversity have been reported (Koren et al., 2012). The dominant phyla in the GM in late pregnancy have been reported to be *Bacteroidetes*, *Firmicutes*, *Proteobacteria*, *Actinobacteria,* and *Verrucomicrobia*, similar to those in early pregnancy (Mokkala et al., 2021).

Many studies have demonstrated the beneficial effects of exercise (Febbraio, 2017; Garber et al., 2011). On the other hand, the development of most chronic diseases, such as obesity (Wan et al., 2020), diabetes (Mojsak et al., 2020), inflammatory bowel disease (Zuo & Ng, 2018), and autoimmune diseases (Marietta et al., 2019) is associated with the GM. A previous study found that exercise can promote health and treat many chronic diseases by changing the structure of GM (Monda et al., 2017). Regular exercise can increase GM diversity and the relative abundance of beneficial bacteria in the gut (Barton et al., 2018; Clarke et al., 2014; Petersen et al., 2017). However, studies investigating the effects of maternal exercise on the GM during gestation are limited, and mostly involve animal models. A previous study found that wheel-running exercise had no significant effect on the GM of dams fed an HFD and their offspring. However, exercise decreased the α-diversity of GM and altered the relative abundance of bacterial taxa in the offspring of dams fed a chow diet (Bhagavata Srinivasan et al., 2018). A subsequent study also demonstrated that exercise during pregnancy was effective in alleviating metabolic disorders in pregnant mice fed a high-fat diet (HFD) (Chung et al., 2019) and in the offspring of murine dams or sires fed an HFD (Laker et al., 2021), but that it had no significant effect on the composition of the GM (Chung et al., 2019).

However, the exercise interventions in previous experiments were performed by subjecting mice to voluntary wheel exercise during pregnancy, and the amount of exercise gradually decreased as pregnancy progressed, due to the decreased willingness to exercise. In particular, the amount of exercise significantly decreased in pregnant mice in the third trimester, which differed among individuals. Thus, it is difficult to reflect the effect of voluntary exercise on pregnant mice accurately.

Therefore, this study aimed to explore the effects of a set amount of regular maternal exercise during gestation on the GM. For ethical and safety reasons, trials of different diets and exercise cannot be performed on pregnant women. Consequently, we evaluated the effects of swimming exercise on the GM of pregnant mice fed different diets.

## Materials & Methods

### Animals and study design

The study was approved by the Experimental Animal Ethics Committee of Guangdong Medical Laboratory Animal Center (B201808-9), and the experimental animal use license number was SYXK (Cantonese) 2013-0002.

**Figure 1:**
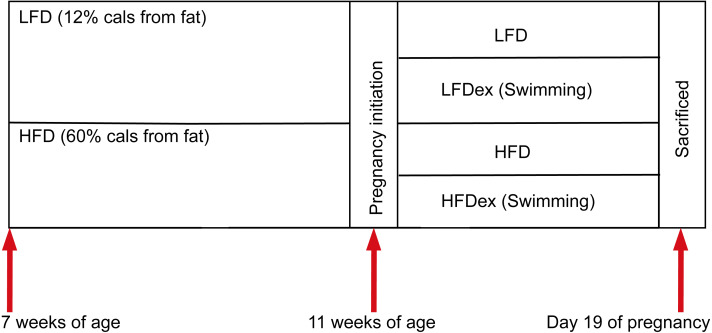
Experimental scheme. Mice were obtained at six weeks of age. After seven days of adaptive feeding, mice were randomly divided into two groups: (1) mice fed on LFD (*n* = 11), (2) mice fed on HFD (*n* = 9). Diet intervention was started at seven weeks of age. After four weeks on the different diet, mice were randomly divided into two groups respectively: (1) remaining sedentary (group LFD, *n* = 5), (2) swimming 10 min a day(group LFDex , *n* = 6), (3) remaining sedentary (group HFD, *n* = 4), (4) swimming 10 min a day (group HFDex , *n* = 5). Exercise intervention in LFDex and HFDex groups were started at 11 weeks of age. The LFDex and HFDex mice were subjected to swimming exercise through gestation until delivery. Colon contents were collected at gestation day 19. LFD, low-fat diet; LFDex, low-fat diet plus exercise; HFD, high-fat diet; HFDex, high-fat diet plus exercise.

Six-week-old specific pathogen-free (SPF) nulliparous female KunMing mice were obtained from the Guangdong Medical Laboratory Animal Center. Mice were maintained in an SPF environment (constant temperature (22–26 °C), humidity (40–70%), and 12-h light/dark cycle) with free access to food and sterile water. The experimental scheme is shown in Fig. 1.

Female mice were randomly divided into two groups after 1 week of acclimation (block randomization was performed according to body weight of mice), according to diet intervention (at 7 weeks of age). One group of mice were fed a low-fat diet (LFD; *n* = 11; 12% fat, 21% protein, 67% carbohydrate), while the other group of mice were fed an HFD (*n* = 9, 60% fat, 20% protein, 20% carbohydrate). The nutritional composition is shown in Table 1.

After 4 weeks on different diets, female mice were mated (with males fed standard chow). Mating was confirmed after the presence of plugs in the female’s vagina or feeding tank. Once pregnancy was confirmed, the day of pregnancy was recorded as GD0. Then, at 11 weeks of age, each group of female mice were randomly divided into two subgroups: a sedentary and an exercise group (swimming for 10 min per day). Thus, mice were divided into group LFD (*n* = 5), group LFDex (*n* = 6); group HFD (*n* = 4), and group HFDex (*n* = 5). The swimming program was performed in a circular water maze (120 cm (diameter), 50 cm (height)) at 24–26 °C with a thermostat used to maintain the water temperature (water depth 30 cm). The water depth was 30 cm so that mice could not support themselves by touching the bottom with their feet. Mice were subjected to swimming in groups and were toweled dry and kept warm after swimming. The protocol was performed at the same time every day (between 9:00 and 11:00 a.m.). LFDex and HFDex mice were subjected to swimming exercise daily, from 11 weeks of age, through gestation, until delivery.

**Table 1:** Ingredient composition of the diets.

**LFD**			**HFD**	
**Ingredients**	g/kg diet		**Ingredients**	g/kg diet
Corn	335.75		Casein	200
Wheat Flour	250		L-Cystine	3
Beancounter	175		Corn Starch	0
Wheat Secondary Flour	133		Maltodextrin	125
Fish Meal	42		Sucrose	68.8
Soybean Oil	25		Cellulose	50
Calcium Bicarbonate	2		Soybean Oil	25
Calcium Carbonate	13		Lard	245
Salt	3		Mineral Mix S10026	10
Choline Chloride	2		DiCalcium Phosphate	13
Mixed Minerals	0.8		Calcium Carbonate	5.5
Vitamins for mice	0.35		Potassium Citrate,1H_2_O	16.5
Vitamin E	0.1		Vitamin Mix 93G	10
Total	1000		Choline Bitartrate	2
			Yellow Dye	0.04
			Blue Dye	0.01
			Total	773.85
Fatty acids	Mass percentage (%)	Energy percentage (%)	Mass percentage (%)	Energy percentage (%)
Saturated fatty acids	0.7	1.86	12.985	22.26
Monounsaturated fatty acids	1.2	3.18	16.1	27.6
Polyunsaturated fatty acids	2.6	6.96	5.915	10.14
Kcal/g	3.51		5.24	

**Notes.**

Ingredient amounts was expressed as g/kg to total 1000.

LFD low-fat diet HFD high-fat diet

During gestation, food intake and body weight of the mice were regularly recorded. On GD19, cervical dislocation was performed for all pregnant mice after isoflurane inhalation anesthesia. Colon contents were collected on GD19 and immediately frozen at −80 °C. All fecal samples were transferred to liquid nitrogen and sent to Novogene (https://www.novogene.com/) for 16S rDNA amplicon sequencing, in the same batch.

### Fecal DNA extraction and gene sequencing

Microbial DNA was extracted from colon contents using the TIANGEN Magnetic Soil and Stool DNA Kit (Qiagen, Hilden, Germany). Fecal DNA was extracted according to the manufacturer’s instructions. Briefly, 0.25−0.5 g of sample or 200 µl if liquid was added to a 2 ml centrifuge tube, and added 500 µl of buffer SA, 100 µl of buffer SC and 0.25 g of grinding beads, vortex mixed, then heat lysis at 70 °C for 15 min to improve lysis efficiency. Lysate was centrifuged for 1 min at 12,000 rpm. About 500 µl of the supernatant was transferred to a fresh 2 ml tube, followed by adding 200 µl of buffer SH, and vortex mixed for 5 min and incubated for 4 min at 4 °C. After centrifuging for 3 min at 12000 rpm, the supernatant was transferred to a fresh 2 ml tube, followed by adding 500 µl of buffer GFA , 10 µl magnetic beads suspension G, and inverted mixed for 5 min. Tubes were placed on the magnetic frame for 30 s, and the solution was removed. The magnetic beads were added with 700 µl RD solution to wash the residual proteins. The magnetic beads were washed with PWD solution two times, then were put to dry at room temperature for 5–10 min. 50–100 µl elution buffer was added and incubated for 5 min at 56 °C to elute the DNA.

We amplified the V3 and V4 regions of the 16S rRNA genes using specific primers with barcodes (Phusion^®^ High-Fidelity PCR Master Mix with GC Buffer; New England Biolabs Corp., Ipswich, MA, USA). Amplicons were quantified using quantitative polymerase chain reaction and a Qubit 2.0 fluorometer (Thermo Fisher Scientific, Waltham, MA, USA). The tags were sequenced using an Illumina NovaSeq 6000 platform (Illumina, San Diego, CA, USA). The reads were merged using FLASH (Version1.2.7, http://ccb.jhu.edu/software/FLASH/) (Magoc & Salzberg, 2011). Reads were analyzed by quality filtering (Bokulich et al., 2013), referring to Quantitative Insights Into Microbial Ecology (QIIME, V1.9.1, http://qiime.org/scripts/split_libraries_fastq.html) (Caporaso et al., 2010).

### Tags quality control process

We used the Uparse software (Version 7.0.1001, http://www.drive5.com/uparse/) (Haas et al., 2011) to cluster all effective tags of all samples. Sequences were clustered into operational taxonomic units (OTUs) with 97% identity by default. The SILVA138 small subunit rRNA database of (http://www.arb-silva.de/) (Edgar, 2013) was used to annotate taxonomic information.

### Statistical and microbial analyses

Statistical analyses, such as diversity measurement, were performed using SPSS software (Version 20.0, IBM Corp, Armonk, NY, USA). Data are expressed as the mean ± standard deviation, and a *t*-test was used to compare the means of the two groups. The least significant difference test was performed for multiple comparisons among multiple groups. Alpha and beta diversity analyses were performed using QIIME software (Version 1.9.1) and R software (Version 2.15.3; https://www.r-project.org/). For alpha diversity, the Chao1 and Simpson indices were analyzed. For beta diversity, principal coordinates analysis (PCoA) plots were constructed based on the weighted UniFrac algorithm distance. Furthermore, linear discriminant analysis (LDA) of the effect size (LEfSe) was used to determine differences between groups. A cladogram depicted an LDA score of 4.0 or 3.0. Phylum- and genus-level top-10 classification comparisons were conducted using the Wilcoxon test with Benjamini and Hochberg false discovery rate correction (FDR-corrected *P*-values represented as q). Statistical significance was set at *P* < 0.05.

## Results

### Characteristics of the study cohort

After mating, five mice in the LFD, six in the LFDex, five in the HFD, and five in the HFDex groups were confirmed to be pregnant. However, a further four female mice were added to the LFDex group as pregnancy was not successfully established(empty pregnancy was found on GD 19). Eventually, five, six, four, and five females in the LFD, LFDex, HFD, and HFDex groups, respectively, successfully conceived. No significant difference was found in gestational weight gain (from GD0 to GD 19) between the two dietary groups. Food intake during pregnancy was slightly lower in the LFDex group than in the LFD group; nevertheless, there were no significant differences in food intake, protein intake, fat intake, and total energy intake between the LFD and LFDex groups. For mice fed an HFD, total energy intake, food intake, protein intake, and fat intake in the HFDex group were significantly lower after exercise intervention than for those in the HFD group (*P* = 0.005, 0.007, 0.003, and 0.001, respectively) (Table 2). The pregnancy success rate, physical characteristics of the pregnant mice, macronutrient intake, and total energy intake in each group are shown in Table 2.

**Table 2:** Physical characteristics of pregnant mice and macronutrient intake and total energy intake.

Groups	Pregnancy success rate^a^	Weight on GD0 (g)	Weight on GD19 (g)	Weight gain during pregnancy (g)	Food intake during pregnancy (g)	Carbohydrates intake (g)	Fat intake (g)	Protein intake (g)	Total energy intake during pregnancy (kcal)
LFD (*n* = 5)	100%	39.28 ± 2.32	75.66 ± 7.88	36.38 ± 7.97	155.58 ± 5.79	91.64 ± 3.41	7.16 ± 0.27	28.78 ± 1.07	546.09 ± 20.31
LFDex (*n* = 6)	60%	37.73 ± 1.44	75.32 ± 11.67	37.58 ± 12.52	152.4 ± 23.07	89.76 ± 13.59	7.01 ± 1.06	28.19 ± 4.27	534.92 ± 80.98
HFD (*n* = 4)	80%	37.93 ± 4.28	69.5 ± 9.10	31.58 ± 11.66	159.98 ± 33.28	42.07 ± 8.75	55.83 ± 11.61	41.91 ± 8.72	838.27 ± 174.37
HFDex (*n* = 5)	100%	39.80 ± 5.46	71.5 ± 5.46	31.7 ± 0.96	116.06 ± 16.11^*^	30.52 ± 4.24	40.5 ± 5.62^*^	30.4 ± 4.22^*^	608.15 ± 84.44^*^

**Notes.**

a The ratio of successfully pregnant mice to all mice identified as entering GD0.

group HFDex compared with group HFD.

* *P* < 0.05 was considered to be statistically significant.

LFD low-fat diet LFDex low-fat diet plus exercise HFD high-fat diet HFDex high-fat diet plus exercise GD gestation day

### Effect of exercise on gut microbiota of mice

**Figure 2:**
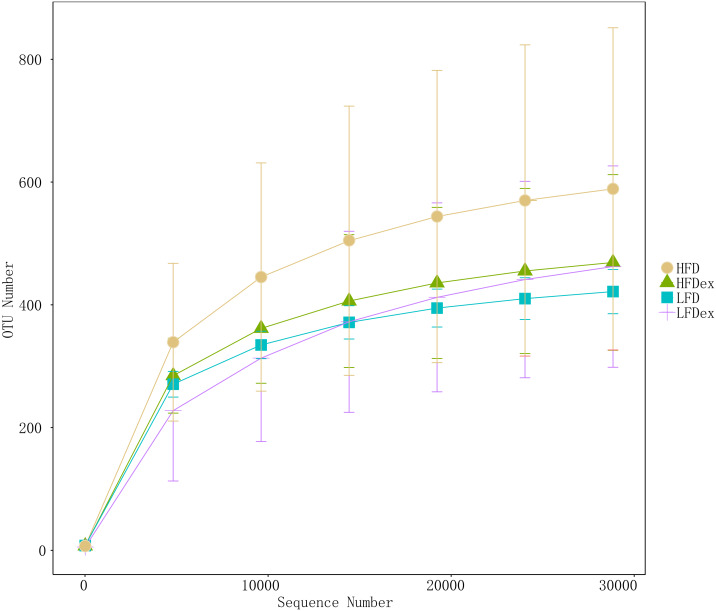
The rarefaction curves of gut samples indicated that the sequencing depth was sufficient. LFD, low-fat diet; LFDex, low-fat diet plus exercise; HFD, high-fat diet; HFDex, high-fat diet plus exercise.

**Figure 3:**
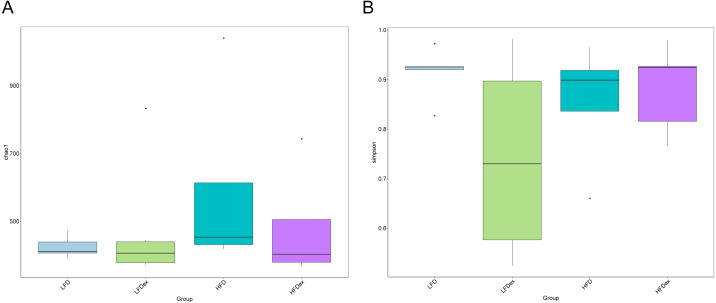
Impact of exercise on gut microbiome. Measures of chao1’s diversity index (A) and Simpson’s diversity index (B) showed no difference when LFD *vs.* LFDex or HFD *vs.* HFDex. LFD, low-fat diet; LFDex, low-fat diet plus exercise; HFD, high-fat diet; HFDex, high-fat diet plus exercise.

To analyze the effect of exercise on the GM, we performed 16S rDNA amplicon sequencing on colon content samples collected from mice on GD19. An average of 28,840 optimized reads were generated from all samples. Rarefaction curves generated from the OTUs in Fig. 2 indicated that the sequencing depth was sufficient to detect all OTUs. The averaged number of OTUs were 936, 1804, 1677, 1326 in the LFD, LFDex, HFD, and HFDex groups, respectively. We first examined two α-diversity indices across the two groups fed different diets. Neither Chao1 nor Simpson indices were significantly different between mice in the LFD group and those in the LFDex group (*P* = 0.971 and 0.180, respectively), HFD group *vs.* the HFDex group (*P* = 0.798 and 0.990, respectively), or between the LFDex group and the HFDex group (*P* = 0.999 and 0.329 respectively), as shown in Figs. 3A and 3B.

**Figure 4:**
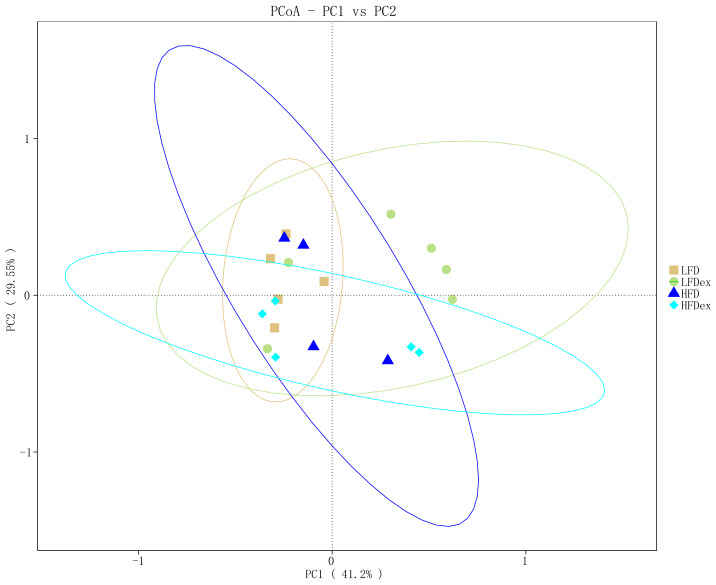
The beta-diversity on weighted UniFrac of gut microbiota composition was assessed by principal coordinates analysis. LFD, low-fat diet; LFDex, low-fat diet plus exercise; HFD, high-fat diet; HFDex, high-fat diet plus exercise.

To determine whether the overall GM composition after exercise interventions under different diets varied, we examined the β-diversity measures. As shown in Fig. 4, PCoA plots based on weighted UniFrac algorithm distance showed that, for mice fed an LFD, the GM communities in the LFDex group were significantly separated from those in the LFD group on the PC1 axis at GD 19, indicating that the structure of the GM was altered in term pregnancy after exercise intervention during pregnancy. For mice fed an HFD, although the GM structure in term pregnancy was generally segregated from that in the HFDex, there was still some aggregation, and the dispersion in the GM between these groups was not as high as that between mice in the LFD and LFDex groups. These results suggest that, although exercise intervention could change the structure of the GM to some extent during pregnancy in mice fed an HFD, the degree of change in the GM structure was not as marked as that in mice fed an LFD. For mice intervened with exercise on differnet diets, the GM structure in term pregnancy was generally segregated in the LFDex *vs.* the HFDex group. These results suggest that the response of exercise intervention to differnet diets may be different.

**Figure 5:**
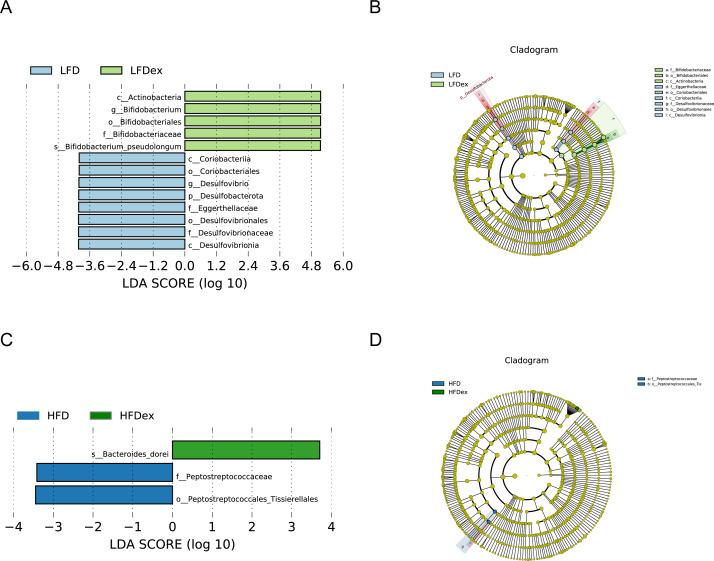
The impact of exercise on gut microbiota composition of mice fed on different diets. The linear discriminant analysis effect size (LEfSe) plotted with threshold for LDA score 4.0 (A and B) showed significant structural difference between the LFD and LFDex groups. Blue bar means taxa significantly increased in group LFD and green bar means taxa significantly increased in group LFDex. The LEfSe plot with threshold for LDA score 3.0 (C and D) showed significant structural difference between the HFD and HFDex groups. Blue bar means taxa significantly increased in group HFD and green bar means taxa significantly increased in group HFDex. LFD, low-fat diet; LFDex, low-fat diet plus exercise; HFD, high-fat diet; HFDex, high-fat diet plus exercise.

**Figure 6:**
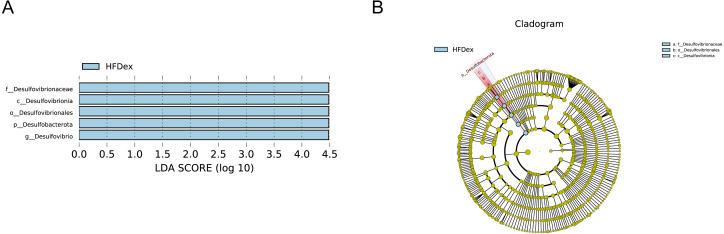
The impact of exercise on gut microbiota composition of mice fed on different diets. The linear discriminant analysis effect size (LEfSe) plotted with threshold for LDA score 4.0 (A and B) showed significant structural difference between the LFDex and HFDex groups. Blue bar means taxa significantly increased in group HFDex.

**Figure 7:**
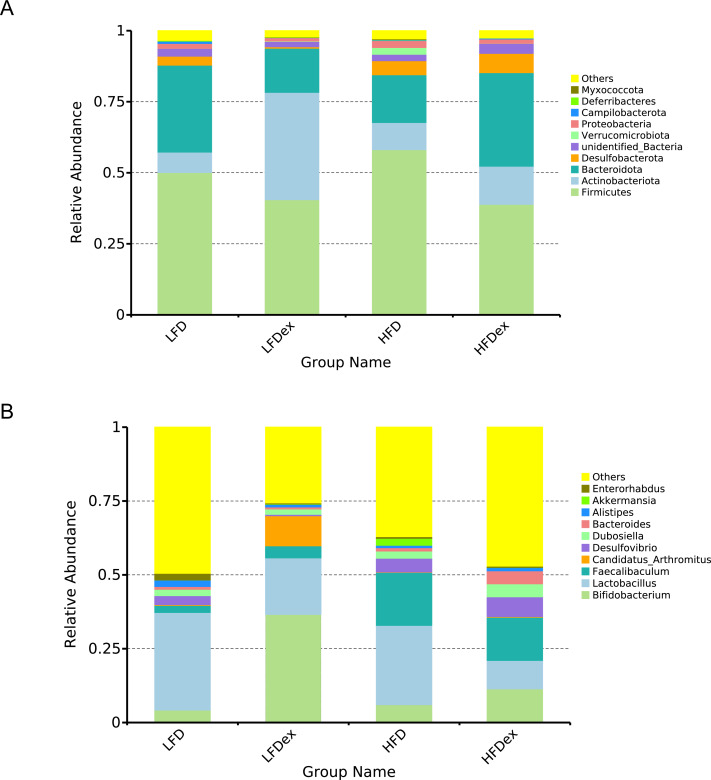
Phylum-(A) and Genus -level (B) top ten classifications in the colon contents of pregnant mice among groups. LFD, low-fat diet; LFDex, low-fat diet plus exercise; HFD, high-fat diet; HFDex, high-fat diet plus exercise.

Next, we performed LEfSe analysis to identify individual GM taxa that differed between sedentary and exercised mice fed different diets. This analysis (LDA score 4.0) revealed that for mice fed an LFD, *p_Desulfobacterota, c_Desulfovibrionia, o_Desulfovibrionales, f_Desulfovibrionaceae and g_Desulfovibrio* were markedly decreased after exercise intervention in the LFDex group as compared to the LFD group. In addition, we observed a marked decrease in *c_Coriobacteriia, o_Coriobacteriales, and f_Eggerthellaceae* in the LFDex group than in the LFD group. However, we found that *c_Actinobacteria*, o_*Bifidobacteriales*, *f_Bifidobacteriaceae, g_Bifidobacterium, and s_Bifidobacterium pseudolongum* were significantly increased after exercise intervention in the LFDex group as compared to that in the LFD group (Figs. 5A and 5B). For mice fed an HFD, when the LDA score was set to 4.0, no significant differences were found in the GM in late pregnancy. Thus, LDA scores of 3.0 were set to identify individual GM taxa that differed between the HFD and HFDex groups. LEfSe analysis revealed that o_*Peptostreptococcales-Tissierellales* and *f_Peptostreptococcaceae* were decreased in the HFDex group as compared to the HFD group. In contrast, s_*Bacteroides_dorei* was more abundant in the HFDex group after exercise intervention than in the HFD group (Figs. 5C and 5D). For mice intervened with exercise on differnet diets, the LEfSe analysis revealed that *p_Desulfobacterota, c_Desulfovibrionia, o_Desulfovibrionales, f_Desulfovibrionaceae and g_Desulfovibrio* were markedly decreased in the LFDex group as compared to the HFDex group (Figs. 6A and 6B), similar to the results in the LFDex group *vs.* the LFD group. The top six dominant phyla in the mice were *p_Firmicutes, p_Bacteroidota, p_Desulfobacterota, p_Actinobacteriota, p_Proteobacteria, p_Verrucomicrobiota* (Fig. 7).

## Discussion

Exercise is now widely accepted as an effective lifestyle factor that can regulate the composition of the GM, maintain its homeostasis, and thus promote the health of the body (Motiani et al., 2020). A previous study (Huang et al., 2016) pointed out that rodents have a congenital swimming ability and that their swimming exercise capacity can be affected by different swimming activity intensities. In this study, swimming for 10 min a day was a very low-intensity exercise that was tolerable for mice and that could be considered as a mimic for exercise during pregnancy. In this study, mice fed an LFD, after 10 min of daily swimming exercise during pregnancy, showed no statistically significant difference in food intake and weight gain during pregnancy as compared to their sedentary counterparts, although these showed a slight increase in food intake. In contrast, in HFD-fed mice, exercise intervention resulted in a significant reduction in maternal food intake as compared to the sedentary group, and the total energy and macronutrient intake during pregnancy in the HFDex group were significantly reduced, but the maternal weight gain was not statistically significantly different from that of the HFD group. It is suggested that the exercise intervention during pregnancy led to a decrease in appetite in mice fed an HFD, for reasons that require further investigation. However, exercise during pregnancy in the HFDex group altered the GM structure and there was no significant decrease in body weight gain during pregnancy as compared to the HFD group.

An interesting phenomenon observed in this study was the effect of exercise on pregnancy success. The traditional Chinese view is that pregnant women should rest during pregnancy to avoid miscarriage. In this study, when designing the exercise during pregnancy, we were also concerned about the increase in miscarriage and preterm birth rates due to exercise during pregnancy. Previous studies (Bhagavata Srinivasan et al., 2018; Chung et al., 2019) have mostly used voluntary exercise by the pregnant mice, and noted that the amount of exercise performed voluntarily by the mice decreased with increasing gestational age. To observe the effect of a set amount of regular exercise during pregnancy on the GM on GD19, mice were subjected to 10 min of swimming exercise per day during pregnancy. Interestingly, we found that the pregnancy success rate of mice fed an HFD and performing 10 min of swimming exercise per day was 100%, whereas mice fed an LFD along with this level of exercise achieved a pregnancy success rate of only 60%, although there was no statistically significant difference between the two groups. Clearly, mice swimming for 10 min had different tolerances to different diets during pregnancy. Therefore, four female mice in the second batch in the LFDex group were supplemented to investigate the effect of an LFD on the GM during gestation. The results of this study seem to indicate that the choice of exercising may be integrated with the adjustment of dietary composition. Whether pregnant women on LFD are also intolerant to exercise during pregnancy? Should the proportion of fat in the diet probably be appropriately increased for pregnant women who choose to exercise during pregnancy? Specific dietary recommendations for pregnant women warrant further investigation.

For mice fed an LFD, although there were some differences in the structure of the GM during late pregnancy between the LFD and LFDex groups, the diversity was not significantly altered by exercise in mice, which is consistent with the findings of a previous study (Bhagavata Srinivasan et al., 2018). The abundance of *p_Actinobacteriota* in the GM of mice in the LFDex group increased significantly, whereas the abundance of both *p_Firmicutes* and *p*_*Bacteroidota* decreased, and the ratio of *Firmicutes*/*Bacteroidota* increased. A previous study showed that exercise increased the abundance of *p_Bacteroidota* in the intestine, thus decreasing the ratio in non-pregnant mice (Mika et al., 2015). Previous researchers found that exercise-induced alterations in the GM could improve glucose homeostasis and insulin sensitivity (Liu et al., 2020). For mice fed HFD, the abundance of *p_Bacteroidota* and *p_Actinobacteriota* in the GM increased in late pregnancy, while the *p_Firmicutes* decreased and the ratio of *Firmicutes*/*Bacteroidota* decreased. Our results are supported by those of previous studies (Bhagavata Srinivasan et al., 2018; Chung et al., 2019). A previous study showed that HFD increased gut microbiota diversity and short-chain fatty acids (SCFAs), causing an increase in energy metabolism (Wang et al., 2020), which was not consistent with our results that the Chao1 and Simpson indices did not altered in mice fed on HFD after swimming intervention.

Analysis of LEfSe revealed that the abundance of *Desulfovibrio* spp. (phylum to genus level) and *Eggerthellaceae* spp. (class to family level) in the GM during late pregnancy decreased significantly after exercise intervention, whereas the abundance of *Bifidobacterium pseudolongum* (class to species level) among *f*_*Actinobacteriota* increased significantly in the LFDex group as compared to the LFD group. Bifidobacteria are important beneficial bacteria in the GM that are involved in sugar metabolism by breaking down complex carbohydrates (Turroni et al., 2018). Their relative abundance in the GM decreases with increasing age of the organism (Mueller et al., 2006). A previous study found that the abundance of *g_Bifidobacterium* in the GM of healthy people who were sedentary was reduced as compared to that in an exercise group (Bressa et al., 2017), which agreed with our results. *Desulfovibrio* spp. is a major member of the sulfate-reducing bacteria, which can produce endogenous hydrogen sulfide, thereby damaging intestinal epithelial cells, and is a class of conditionally pathogenic bacteria. The abundance of *Desulfovibrio* spp. has been shown to be significantly increased in the intestines of patients with allergic purpura and children with autism (Finegold, 2011). *Eggerthellaceae* spp. may be associated with allergic diseases: a recent study (Aparicio et al., 2020) found a higher abundance of this family in the GM of infants allergic to milk protein. When the screening value of the LDA score was set to 4, no significant changes were found at species level in the GM of mice fed an HFD in late pregnancy after exercise intervention. However, when the screening value of the LDA score was reduced to 3, the abundance of *o_Peptostreptococcales-Tissierellales* and *f_Peptostreptococcaceae* decreased and that of *s*_*Bacteroides dorei* increased in the late pregnancy GM of mice in the HFDex group as compared to that of the HFD group. Therefore, our results indicate that, for mice on an HFD, exercise during pregnancy also altered the structure of the GM in late pregnancy to some extent; however, the effect was smaller than that in mice fed an LFD.

An early study demonstrated that proper exercise during pregnancy can improve the metabolism of pregnant women and prevent a variety of pregnancy complications, such as obesity, with multiple benefits for the mother and newborn (Laker et al., 2021; Zhou et al., 2020). Studies have also shown that exercise during pregnancy can reduce the adverse effects of obesity in the mother and newborn (Mourtakos et al., 2015; Weissgerber et al., 2006). In the present study, we investigated the effect of exercise during pregnancy on the GM in mice fed various diets. Our results showed that, for mice fed an LFD, exercise during pregnancy did not change the diversity of the GM in GD19 but altered the structure of the GM to some extent, increasing the abundance of beneficial bacteria and decreasing the abundance of conditionally pathogenic bacteria in the intestines of the mice. For mice fed an HFD, exercise intervention did not alter the diversity of the GM in late pregnancy, but it did alter the abundance of some species in the GM to some extent, improving the structural changes in the GM induced by HFD. Our study revealed that shifts in GM composition by exercise intervention were diet-dependent, contradicting a previous study that showed that most diet- or exercise-induced alterations in GM populations were unrelated (Kang et al., 2014). Owing to the influence of the traditional Chinese concept of fetal preservation, most pregnant women exercise very little during pregnancy. Based on the results of this experiment, it is recommended that pregnant women perform appropriate exercise during pregnancy, particularly those who have consumed an HFD for a long time. In pregnant women who consume a high-carbohydrate diet, exercise can be appropriately reduced during early pregnancy.

The pregnancy success rate of mice in the LFDex group was significantly lower than that of mice in the other groups. This discrepancy may be related to the fact that pregnancy did not succeed after intercourse or that miscarriage occurred in early pregnancy. However, little is known about the specific reasons for this situation. Since both GM and pregnancy are closely related to the immune status of the body, if the immune status is imbalanced, it will cause the individual to be less likely to conceive or to have an early abortion after conception. Therefore, we postulate that exercise intervention during pregnancy might affect pregnancy success by affecting GM and thus organismal immunity in mice fed LFD. No relevant studies on this topic are available, and the reason for the significantly lower pregnancy success in the LFDex group of mice still requires further investigation. Our results indicate that exercise during gestation is likely to benefit people eating an HFD. Therefore, it may be appropriate to increase the proportion of fat in the diet of pregnant women who choose exercise interventions during pregnancy, but specific exercise recommendations warrant further investigation.

Our study had some limitations. First, despite the small sample size, the results of this animal experiment were plausible due to the uniformity of genetic strains and interventions, which differed from human experiments. Although the number of cases was small, our investigation showed significant differences. Second, although the experimental protocols were the same, we could not exclude potential confounding factors because different batches of mice were used. Third, given that the dietary source in LFD and HFD was considerably different, this could also affect gut microbiota compositions, apart from high/low fat composition.

## Conclusions

A quantitative exercise intervention during pregnancy increased the abundance of beneficial bacteria and decreased the abundance of conditionally pathogenic bacteria in the gut of pregnant mice fed an LFD, although the pregnancy success rate was significantly decreased. In mice on an HFD, exercise intervention during pregnancy significantly reduced the *Firmicutes/Bacteroidota* ratio in pregnant mice, and mice still maintained a high pregnancy success rate after exercise intervention. Our results indicated that exercise during gestation induced changes in maternal GM. These data may have important implications for considering an individual’s overall diet when recommending exercise during pregnancy.

##  Supplemental Information

10.7717/peerj.14459/supp-1 Supplemental Information 1 Physical characteristics of pregnant mice and macronutrient intake and total energy intake

10.7717/peerj.14459/supp-2 Supplemental Information 2 OTU relative abundance table

10.7717/peerj.14459/supp-3 Supplemental Information 3 Phylum level relative abundance table

10.7717/peerj.14459/supp-4 Supplemental Information 4 ARRIVE 2.0 Checklist

10.7717/peerj.14459/supp-5 Supplemental Information 5 Genus level relative abundance table

## Abbreviations

GD: gestation day
GM: gut microbiota
HFD: high-fat diet
HFDex: high-fat diet plus exercise
LDA: linear discriminant analysis
LEfSe: linear discriminant analysis of the effect size
LFD: low-fat diet
LFDex: low-fat diet plus exercise
OTU: Operational Taxonomic Units
PCoA: principal coordinates analysis

## Additional Information and Declarations

### DNA Deposition

The following information was supplied regarding the deposition of DNA sequences: The original sequencing data are available in NCBI: PRJNA844908.

### Data Availability

The following information was supplied regarding data availability: The raw data are available in the Supplementary Files.
